# Helium Ion Microscope-Assisted Nanomachining of Resonant Nanostrings

**DOI:** 10.3390/s16071080

**Published:** 2016-07-13

**Authors:** Wei Zheng, Peng Li, Remko van den Hurk, Stephane Evoy

**Affiliations:** 1 Department of Electrical and Computer Engineering, University of Alberta, Edmonton, AB T6G 1H9, Canada; wzheng2@ualberta.ca (W.Z.); remko@ualberta.ca (R.v.d.H); 2 Nanofab, University of Alberta, Edmonton, AB T6G 1H9, Canada; peng.li@ualberta.ca

**Keywords:** helium ion, milling, nanostrings, resonators, nanoelectromechanical systems

## Abstract

Helium ion microscopy has recently emerged as a potent tool for the in-situ modification and imaging of nanoscale devices. For example; finely focused helium ion beams have been used for the milling of pores in suspended structures. We here report the use of helium ion milling for the post-fabrication modification of nanostrings machined from an amorphous SiCN material. The modification consisted of milling linear arrays of holes along the length of nanostrings. This milling results in a slight decrease of resonant frequency while increasing the surface to volume ratio of the device. The frequency decrease is attributed to a reduction of the effective Young’s modulus of the string, which in turn reduces the tension the string is under. Such experimental observations are supported by the finite element analysis of milled and non-milled strings.

## 1. Introduction

The assaying of biological targets is of significant importance for biomarker analysis, molecular assays, and drug development. Conventional platforms such as nuclear magnetic resonance, mass spectrometry, enzyme-linked immunosorbent assay (ELISA) are however not suitable for the analysis of large number of analytes [1]. Antibody microarrays [2] represent a potent alternate option for such mutiplexed assays. However, traditional detection approaches such as chemiluminescence, colorimetry and fluorescence require a second labelled antibody in order to provide the required specificity and sensitivity.

Label-free biosensors offer alternatives for such multiplexed assays [3,4]. A biosensor traditionally integrates a transduction platform and a molecular probe, along with analysis and read-out circuitry. For example, micromechanical resonant sensors function by monitoring changes of resonant frequency induced by the binding of a target analyte [5]. This method allows the frequency modulation of the output signal, thus enhancing its noise-immunity and stability. Further, the detection sensitivity per unit area of nanoelectromechanical systems favourably as their dimensions are reduced, offering a compelling platform for the development large arrays of devices with high sensitivities. These arrays would enable multiplexed binding assays able to analyze complex mixtures of biomolecules. Resonant nanostrings [6] thus offer a promising platform for array-based detection. Sub 100 nm-wide silicon nanowires were first reported by Carr Evoy et al. [7]. These devices were measured using a Fabry-Perot interferometric method developed by the same research group. Brittleness and stiction issues however impaired the yield of these ~50 nm-wide wires to less than 25%. Since that time, a number of alternate “bottom-up” approaches based on controlled assembly [8,9,10,11] or directed growth [12,13,14,15] have been reported. Alternatively, we recently developed a novel “top-down” technique for the realization of nanostrings. As opposed to the now widely-employed silicon-on-insulator approach, this method combines the patterning of an amorphous SiCN layer with the bulk etching of the underlying silicon substrate for the release of the nanostring [16,17]. This process readily enables the fabrication of sub-20 nm wide and tens of micrometers long strings with very high yield [18]. Sub-10 nm wide nanostrings were subsequently realized [19]. This being said, the interferometric measurement of the devices becomes challenging at widths narrower than 20 nm. Alternate approaches are therefore desired in order to produce resonators of high surface-to-volume ratio while retaining their amenability to optical measurement.

Post-fabrication nanostructuring of released mechanical structures has been performed using electron beam writing [20], focused ion beam milling [21,22], track etching [23], and dielectric breakdown [24]. Alternatively, helium ion microscopy (HeIM) has recently emerged as a versatile tool for the in-situ modification and imaging of nanoscale features [25,26,27,28,29,30]. For example, finely focused helium ion beams have been used for the milling of pores in SiN [31,32] and Si [33] membranes. Such accuracy offers interesting possibilities for the tuning of mechanical properties of suspended structures. More specifically, local milling of nanostrings would enable the control of resonant mode shapes and clamping point design [18,34,35]. We thus report the first demonstration of HeIM-nanomachining for the post-fabrication tuning of nanomechanical resonators. The helium ion beam is used to locally mill pores along the length of nanostrings fabricated from an amorphous SiCN material. The effect of this machining on resonant mode shape and frequency is assessed using both finite element analysis and experimental measurements.

## 2. Materials and Methods

### 2.1. Fabrication of SiCN Nanostrings

**Figure 1:**
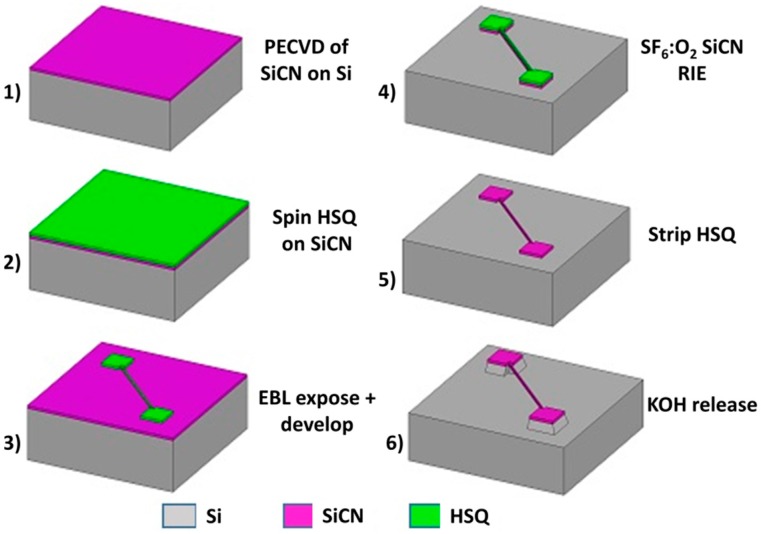
Process diagram for the fabrication of SiCN nanostrings. From [36].

The fabrication of the SiCN nanostrings is described in Figure 1 [19,36]. A (100) silicon wafer (thickness of 500 μm, diameter of 100 mm) was first cleaned in a piranha solution (3:1 H_2_SO_4_:H_2_O_2_) for 15 min and buffered oxide etch (BOE, 10:1 HF:NH_4_F) for 3 min. A SiCN layer of 50 nm thickness was deposited in a plasma enhanced chemical vapor deposition (PECVD) reactor at a pressure of 500 mTorr, a deposition temperature of 300 °C and using a gas ratio of 4:1 NH_3_:diethysilane (DES). A Filmetrics (Filmetrics, San Diego, CA, USA) thickness mapping system was employed to measure the film thickness. The residual stress of the deposited SiCN was measured using a Flexus 2320 wafer stress measurement system (KLA-Tencor, Chandler, AZ, USA). As-deposited films showed a compressive stress of −764 MPa. The wafer was then annealed in a MiniBrute 3 zone tube furnace (Thermco Systems, West Sussex, UK) for 2 h at 525 °C in order to desorb included hydrogen. Following this anneal, the films showed a tensile stress fluctuating between 375 MPa and 450 MPa. The wafer was cut into 0.7 cm × 0.7 cm pieces and cleaned in piranha again prior to the coating of the resist. Hydrogen silsesquioxane (HSQ, XR-1541, Dow Corning, Corning, NY, USA) was employed as electron beam lithography (EBL) resist due to the simplicity involved and its high resolution capabilities. Diluted 1% HSQ was coated onto the dies using using a spin speed of 4000 rpm for 40 s. The resist was then baked at 90 °C for 5 min. The final thickness of the resist was 28 nm.

The resist was then exposed using a Raith 150 *^Two^* EBL instrument (Raith GmbH, Dortmund, Germany) using a 10 μm aperture and a 10 kV acceleration voltage. Area doses ranging between 0.5 and 1.75 mC/cm^2^ were employed to pattern the anchor pads and strings wider than 50 nm. Line doses of 4.9–9.1 nC/cm were employed to pattern narrower nanostrings. Following exposure, the resist was developed in 25% tetramethylammonium hydroxide (TMAH) at room temperature for 75 s. The die was then rinsed in water and dried using nitrogen flow. The SiCN film was etched by reactive ion etching (RIE) in a 4:1 SF_6_:O_2_ plasma using the patterned resist as etch mask. The resist was then removed by a 30 s dip in buffered oxide etch (BOE). Finally, the SiCN nanostrings were released by anisotropic etching of the silicon substrate. This etch was performed through immersion in a 35% KOH solution saturated with IPA at 75 °C for 40–135 s, depending on the string length.

### 2.2. Helium Ion Milling

Helium ion milling of holes on 200 nm-wide nanostrings was performed in a Zeiss Orion NanoFab Helium Ion Microscope (Carl Zeiss AG, Oberkochen, Germany). The milling was performed using an ion beam current of 13.7 pA, a beam energy of 31 keV, and a nominal beam size of 0.5 nm. The patterning was performed with a step size of 1 nm at a dose of 30 nC/m^2^. For a given device, the initial beam alignment took 10 min. The sample positioning and stage settle-down took an additional 10 min. The holes were then patterned successively with a milling time of 5 s per hole. The modification consisted of milling a linear array of holes along the length of the string. The individual holes had a diameter D = 45 nm, with a centre-to-centre spacing of s = 120 nm.

### 2.3. Laser Interferometry Apparatus

**Figure 2:**
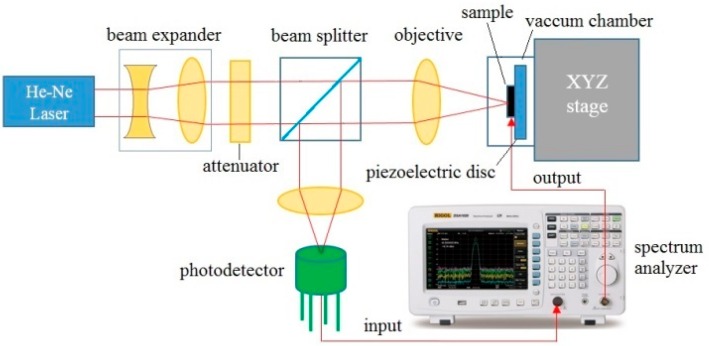
Schematic diagram of interferometric system employed for the measurements of resonant frequency of SiCN nanostrings. From [36].

Resonant frequencies were measured using Fabry-Perot interferometry [12,13]. A schematic diagram of the experimental setup is shown in Figure 2. The chip was affixed onto a piezoelectric actuator and placed in a vacuum cell under a pressure of 10^−3^ Torr. The tracking signal of a spectrum analyzer (Agilent E4411B, Agilent Technologies, Santa Clara, CA, USA) was employed to actuate the piezoelectric disk. A 633 nm He-Ne laser beam was expanded, attenuated, and focused onto the nanostrings using a microscope objective. The vibrating string and substrate form an oscillating Fabry Perot cavity that modulates the reflected signal. The resulting fringe pattern was passed back through the microscope objective, redirected by a beam splitter, and focused on a AC coupled photodetector using a convex lens. The photodetector signal is then sent to the input port of the spectrum analyzer. The resonance was measured at the frequency of largest vibration amplitude.

### 2.4. Finite Element Analysis

Finite element analyses were performed using COMSOL Multiphysics version 4.3 (COMSOL Group, Burlington, MA, USA) in 3D solid, stress-strain mode using its structural mechanics module. The doubly clamped beam was modelled as a rectangular box with dimensions of 15 μm in length, 200 nm in width, and 50 nm in thickness, and clamped at both extremities along the long axis. The material properties employed were a density ρ = 2200 kg/m^3^ and a Young’s modulus of E = 108 GPa, as per experimentally measured values reported in ref. [17]. The intrinsic tensile stress was defined along the longer axis in the linear elastic material model as an initial stress.

For the unmilled beams, a mesh composed of 7703 tetrahedral elements was created using the physics-controlled meshing technique. The eigenfrequency analysis was performed in two steps. Firstly stationary analysis was performed to solve for the effect of initial stress on the resonator. Secondly the frequency response of the resonator was determined through eigenfrequency analysis. Additional simulations performed with 57,117 tetrahedral elements yielded resonant resonant frequencies that were within 0.02% of the one obtained with 7703 elements.

**Figure 3:**
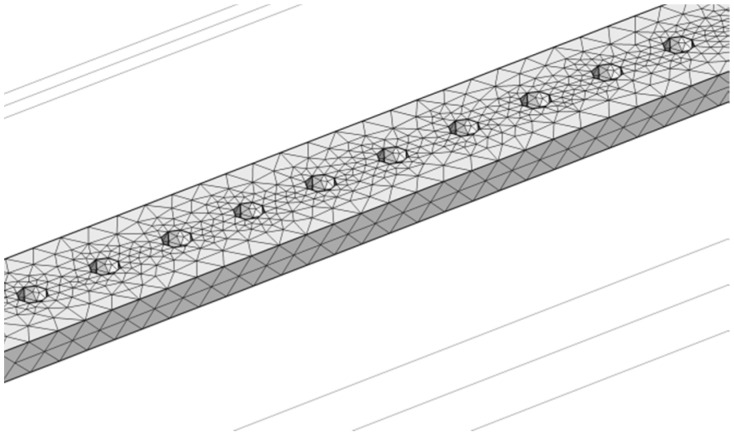
Meshing employed for the finite element analysis of the resonant frequency of SiCN nanostrings of length L = 15 μm, width W = 200 nm and thickness t = 50 nm. The strings are milled with linear array of holes of diameter D = 45 nm and center-to-center spacing s = 120 nm. The figure shows a 1.2 m long segment of the string. The meshing of the whole string employed a total of 92,243 tetrahedral elements.

We also simulated the effect of the milled holes on the calculated resonant frequencies. In those simulations, linear arrays of holes with 45 nm diameter and centre-to-centre spacing of 120 nm were defined along the length of the string. Additional simulations in which the holes were substituted by a hypothetical material of Young’s modulus equal to the one of the SiCN, but with a near-zero density were also conducted. These additional simulations were performed to untangle effects related to mechanical properties from effects related to density. The eigenfrequencies were computed through the aforementioned two steps analysis. Both sets of simulations employed a mesh of 92,243 elements in order to insure accurate rendering of the holes. Additional simulations involving 288,720 elements yielded resonant frequencies falling within 0.1% of those obtained with 92,243 elements. The frequency shifts were calculated with respect to the eigenfrequency of the original resonator. Figure 3 shows a typical meshing used for the simulation of milled beams.

## 3. Results and Discussion

### 3.1. Modelling

The fundamental resonant frequency of a beam of rectangular cross-section, clamped on both ends, under no stress, and vibrating perpendicular to is thickness is given by [37,38]:

(1) $$f_{0}\;=\;1.028\frac{t}{L^{2}}\sqrt{\frac{E}{\rho }}$$

where *E*, *t* and *L* are the Young’s modulus, density, thickness and length, respectively. When a tensile stress is present along the axial direction, the resonant frequency *f_0_* will be changed to a new value *f_1_*:

(2) $$f_{1}\;=\;f_{0}{\left(1\;+\;\frac{0.295\sigma L^{2}}{Et^{2}}\right)}^{\frac{1}{2}}$$

The reader can consult ref [38] for the derivation of Equations (1) and (2). A systematic analysis of clamped-clamped resonators fabricated in this material has been reported in [17]. In that work, clamped-clamped resonators showed a *L^−0.999^* dependence of *f_0_*, suggesting that the devices were operating in the high-stress limit and thus dominated by the second term of Equation (2). The same analysis of singly clamped vs doubly clamped devices inferred a density *ρ* = 2200 kg/m^3^ and a Young’s modulus of *E* = 108 GPa [5]. This being said, this material was also found to present substantial variation of tensile stress within different locations of a given wafer, and from wafer to wafer. In our experiments, the tensile stress each device is under is thus a priori not precisely known.

**Figure 4:**
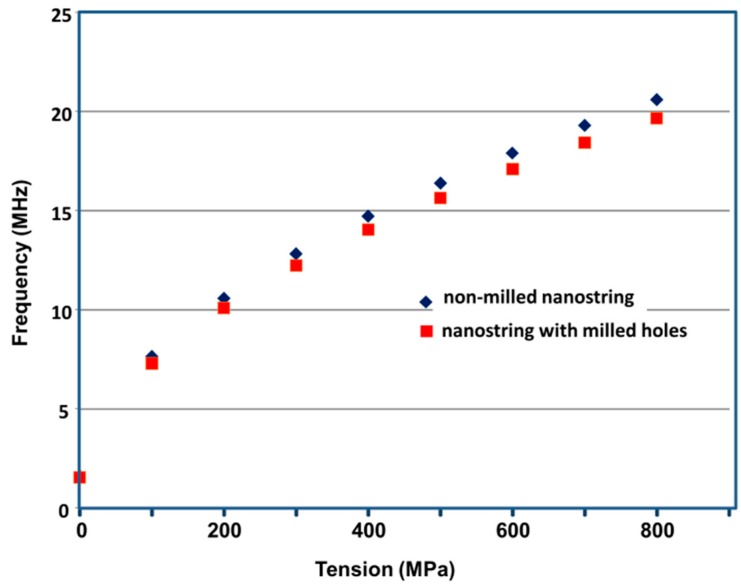
Finite element analysis of of the resonant frequency of SiCN nanostrings of length L = 15 m, width *W* = 200 nm and thickness *t* = 50 nm under varying tensile stress. Results for non-milled strings and strings milled with linear array of holes of diameter *D* = 45 nm and center-to-center spacing s = 120 nm are shown.

The simulated resonant frequency of non-milled nanostrings of width *W* = 200 nm, thickness *t* = 50 nm and length *L* = 15 um as function of tensile stress is found in Figure 4. The resonant frequencies range from *f_0_* = 1.6 MHz for *σ* = 0 MPa to *f_0_* = 20.6 MHz for *σ* = 800 MPa. Once again, the relationship between frequency and stress becomes linear for stresses above 350 MPa, further confirming that the frequency becomes dominated by second term of Equation (2) above those values.

**Figure 5:**
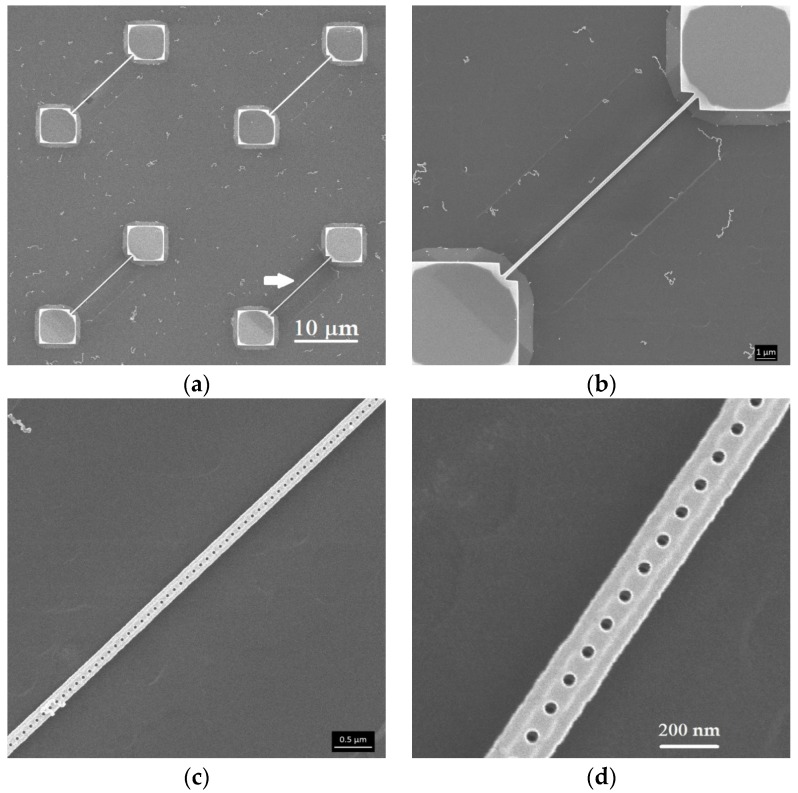
(**a**) Array of nanostrings with helium ion beam-milled holes; (**b**–**d**) High magnifications images of milled nanostring shown by arrow in (**a**).

The simulated resonant frequency of milled nanostrings of similar dimensions is shown on same figure. In these simulations, a linear array of holes of diameter *D* = 45 nm and center to center spacing *s* = 120 nm is included in the modelling. As seen in Figure 5, the presence of the holes reduce the resonant frequency by 4.5% independently of tensile stress.

The presence of holes is expected to affect both the effective mechanical properties of the string (such as Young’s modulus and tension), as it does affect its effective linear density. While a reduction of tension and Young’s modulus would decrease the resonant frequency, a reduction of linear density would rather increase it. To untangle the effects, another set of simulations was conducted in which the holes were rather replaced by a hypothetical material whose Young’s modulus was equal to the one of the SiCN, but whose density was near zero. In that case, the nanostructuring increased the simulated resonant frequency by ~3%. Indeed, by keeping the average Young’s modulus constant, these simulations now solely accounted for the reduction of linear density of the material. From Equation (1), the following approximate relationship is derived:

(3) $$\left|\frac{\Delta f}{f}\right|\;=\;-\frac{1}{2}\left|\frac{\Delta \rho }{\rho }\right|$$

While the volume of the original string equals 150 × 10^6^ nm^3^, the combined volume of the holes totals 9.94 × 10^6^ nm^3^. The holes thus reduce the linear density of the string by 6.7%. Equation (2) would thus predict that such 6.7% reduction would result in a 3.3% increase of resonant frequency, as observed in the simulations.

As mentioned above, the presence of fully voided holes did however result in a net reduction of 4.5% of the frequency, in spite of the 3.3% increase that would result if only change of effective density would be involved. In addition, this 4.5% net relative decrease is independent of tensile stress, thus indicating that the presence of holes affects both the Young’s modulus (first term of Equation (1)) and the axial stress (second term of Equation (2)) equally. This behavior was not unexpected given the relationship existing between Young’s modulus *E*, stress *σ* and strain *ε* :

(4) σ = Eε

Indeed, the milling of the holes is not expected to relieve the beam from the tensile strain *σ* it is under. This being said, the milling of holes effectively reduces the average Young’s modulus *E*, thus in turn affecting the stress *σ* the device is under. Such mechanical effect would offset the effect of reduction of density with the net result of having the string’s resonant frequency be reduced by the milling through reduction of both *E* and *σ*.

### 3.2. Experimental Measurements

Figure 5a shows an array of milled SiCN nanostrings. Figure 5b–d show higher magnification images of the nanostring indicated by the arrow in Figure 5a. The presence of the linear array of milled holes is clearly visible along the length on the structure.

Non-milled and milled devices were then measured using the interferometry system shown in Figure 2. The average resonant frequency of non-milled devices was measured to be 13.5 ± 0.2 MHz. The devices showed a resonant quality of *Q* = 5500 as measured from the full-width at half-maximum. When comparing this result to the simulated values (Figure 4), this corresponds to a tensile stress approximately 375 MPa, within the range usually observed from this material. In turn, the resonant frequencies of the milled devices were measured to be 12.8 ± 0.3 MHz. A ~5% net reduction of frequency is thus observed, as was predicted by the FEA analysis of the devices. The quality of the resonance was not measurably affected by the milling. This being said, this reduction of resonant frequency is accompanied by a net increase of surface-to-volume ratio. Indeed, a 15 m × 50 nm × 200 nm non-milled string possesses a volume of 150 × 10^6^ nm^3^ and a surface area of 7.5 × 10^6^ nm^2^, corresponding to a surface to volume ratio of 0.05 nm^−1^. In turn, the milling of holes with diameter of *D* = 45 nm and center to center spacing of *s* = 120 nm reduces the volume of the beam to 140 × 10^6^ nm^3^, while augmenting its available capture surface to 8.38 × 10^6^ nm^2^. This corresponds to a new surface to volume ratio of 0.06 nm^−1^, a 20% increase compared to the non-milled devices.

## 4. Conclusions

We have reported the use of helium ion milling for the post-fabrication modification of nanomechanical resonators. More precisely, arrays of holes were fabricated along the length of nanostrings. This patterning resulted in a slight reduction of the resonant frequency of the devices, while increasing their surface to volume ratio. Helium ion milling could therefore be used for the post-fabrication tuning and trimming of nanomechanical resonators. This milling technique is highly flexible and offers precise control over the dimension, locations and numbers of the milled patterns.
